# Influence of probiotic supplementation on the developing microbiota in human preterm neonates

**DOI:** 10.1080/19490976.2020.1826747

**Published:** 2020-10-23

**Authors:** Niels van Best, Sonja Trepels-Kottek, Paul Savelkoul, Thorsten Orlikowsky, Mathias W. Hornef, John Penders

**Affiliations:** a Institute of Medical Microbiology, RWTH University Hospital Aachen, RWTH University Aachen, Aachen, Germany; b Department of Medical Microbiology, School of Nutrition and Translational Research in Metabolism (NUTRIM), Maastricht University, Maastricht, The Netherlands; c Section of Neonatology, University Children’s Hospital, Aachen, Germany; d School of Public Health and Primary Care, Maastricht University, Maastricht, The Netherlands

**Keywords:** enteric microbiota, preterm, probiotic supplementation, necrotizing enterocolitis, pathobiont

## Abstract

**Background:**

Oral administration of probiotic bacteria to preterm neonates has been recommended to prevent the development of necrotizing enterocolitis (NEC). The influence of probiotics on the endogenous microbiome, however, has remained incompletely understood.

**Study design & methods:**

Here, we performed an observational study including 80 preterm neonates born at a gestational age <32-weeks to characterize the persistence of probiotic bacteria after no treatment or oral administration of two different probiotic formula and their influence on the microbial ecosystem during and after the intervention and their association with the development of NEC. Weekly fecal samples were profiled by 16S rRNA sequencing and monitored for the presence of the probiotic bacteria by quantitative PCR.

**Results:**

Microbiota profiles differed significantly between the control group and both probiotic groups. Probiotic supplementation was associated with lower temporal variation as well as higher relative abundance of *Bifidobacterium* and *Enterobacter* combined with reduced abundance of *Escherichia, Enterococcus*, and *Klebsiella*. Colonization by probiotic bifidobacteria was observed in approximately 50% of infants although it remained transient in the majority of cases. A significantly reduced monthly incidence of NEC was observed in neonates supplemented with probiotics.

**Conclusion:**

Our results demonstrate successful transient colonization by probiotic bacteria and a significant influence on the endogenous microbiota with a reduced abundance of bacterial taxa associated with the development of NEC. These results emphasize that probiotic supplementation may allow targeted manipulation of the enteric microbiota and confer a clinical benefit. (Clinical Trial Registry accession number: DRKS/GCTR 00021034)

## Introduction

Following birth, establishment of the early intestinal microbiota is characterized by major fluctuations in the relative abundance of the prominent bacterial taxa associated with an increase in the bacterial diversity over time.^1,2^

Preterm birth increases the inter-individual variation in the early fecal microbial ecosystem and enhances the colonization by opportunistic pathogens due to delivery by cesarean section, delayed onset and a reduced rate of enteral feeding by breast milk, the need for antibiotic therapy and prolonged exposure to the hospital environment.^3,4^ Together with the immature immune system and mucosal tissue, these microbiota alterations may enhance the susceptibility for necrotizing enterocolitis (NEC), an inflammatory disease of the preterm neonate’s mucosal tissue associated with high morbidity and mortality.^5^

Oral administration of probiotic bacteria to preterm neonates was reported to decrease the all-cause mortality and in particular the incidence of NEC in a number of studies.^6,7^ Despite these promising results and the subsequent routine probiotic administration to preterm neonates in some institutions,^8,9^ the precise influence of the administered probiotic bacteria on the existing microbial ecosystem during and following the intervention has remained incompletely understood. In particular, this relates to the selection and comparative analysis of probiotic strains and formulations, the persistence of the administered probiotic bacteria as well as their influence on the preexisting colonizing bacteria.^10–12^ To gain more insight into the underlying mechanisms, we compared the microbiota in 174 fecal samples of 80 preterm neonates in the absence of as well as prior to, during and after oral administration of two different probiotic consortia within the context of a natural experiment.

## Results

### Study population characteristics

**Figure 1.**
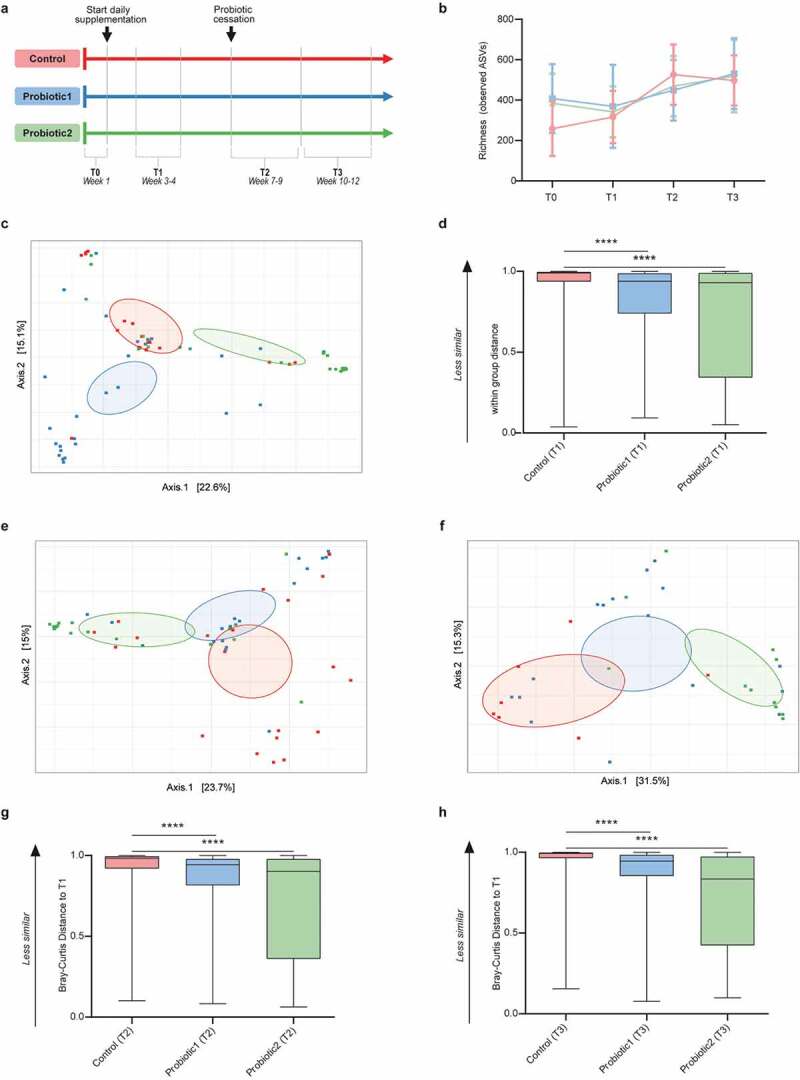
**Microbiota composition and stability in neonates with or without probiotic supplementation**. (a) Infographic of study outline (n = 51–66 per group for all subsequent cohort-analyses) (b) Microbial richness (number of ASVs) shows a gradual increase with age for all groups (linear regression: p_for trend_<0.0001, p_for trend_ = 0.01, p_for trend_ = 0.0021 for controls, probiotic1, probiotic2, respectively). (c) Principal Coordinate Analysis (PCoA) based on ASV-derived Bray-Curtis dissimilarity exhibiting differences in microbial community structure between control and probiotic groups at T1 (*p* < 0.001, Permanova). (d) Bray-Curtis distance compared to T1 at T1 (within group distance). (e,f) PCoA between control and probiotic groups at T2 (*p* < 0.001, Permanova) (e), and at T3 (*p* < 0.001, Permanova) (f). (g,h) Bray-Curtis distance compared to T1 at T2 (g), and at T3 (h). (Kruskal-Wallis test with Dun’s test for post-hoc comparisons (control-group is reference), mean and SD; ****, *p* < 0.0001)

As part of a longitudinal observational study, we collected fecal material of preterm infants hospitalized at the NICU of RWTH Aachen University Hospital weekly between January 2016 and 2018. Between January 2016 and May 2016, neonates born at <32-weeks gestational age received daily probiotic supplementation consisting of *Lactobacillus acidophilus* (ATCC 4356) and *Bifidobacterium longum* subspecies *infantis* (ATCC 15697) subsequently referred to as *B. infantis* as a standard clinical procedure (probiotic 1 [P1] group). In May 2016, this probiotic was no longer commercially available and supplementation had to be ceased (control group). From January 2017 onwards, another probiotic mixture consisting of *Lactobacillus acidophilus* La-14 (ATCC SD5212), *Bifidobacterium longum* subsp. *longum* Bl-05 (ATCC SD5588) subsequently referred to as *B. longum, Lactobacillus casei* Lc-11 (ATCC SD5213), and *Bifidobacterium animalis* subsp. *lactis* (ATCC SD5215) subsequently referred to as *B. lactis* (probiotic 2 [P2] group) was introduced and administered to preterm neonates. Supplementation of both probiotic products continued until 36 weeks postmenstrual age. In total, we selected 174 fecal samples from 80 preterm neonates. The population characteristics, including gestational age, birthweight, sex, and feeding-mode, were comparable between all three groups (Table S1). The median gestational age for preterms in the control, P1 and P2 group was 29, 30, and 26 weeks, respectively, and most neonates were born by cesarian section. For the comparative analysis between groups (P1, P2 and control group), samples were clustered in four age-windows as follows: week 1 after birth, week 3–4 after birth, week 7–9 after birth, and week 10–12 after birth corresponding to “before” (T0), “during” (T1), “shortly after” (T2) and “long after” (T3) probiotic supplementation, respectively (Figure 1a).

### Supplementation of probiotic bacteria affects the microbiota composition in early life

To assess global compositional differences in the gut microbiome of neonates, we profiled fecal samples by V3-V4 16S rRNA gene amplicon sequencing. A total of 7,708,120 sequences with a median of 21,113 [range 7,727–72,816] reads per sample were retained after quality filtering and assigned to 4,981 amplicon sequence variants (ASVs) for subsequent analyses.

The fecal microbial richness gradually increased significantly with age (linear regression *p* < 0.0001, *p* = 0.01, and *p* = 0.0021 for control, P1, and P2 groups, respectively), but was not significantly different between the control and probiotic groups (Figure 1b, Figure S1a-d). Similar results were obtained for the microbial diversity as examined by the Shannon index (Figure S1e-h).

As expected, no significant difference in the general microbial community composition was observed between the three groups prior to the start of probiotic supplementation (T0) (Figure S2a). In contrast, significant differences in the microbial composition between the control group and both probiotic groups at the time of probiotic supplementation was observed (Figure 1c). The changes in the microbial community structure remained after identification and correction for potential confounders such as feeding-type, sepsis, administration of antibiotics and gestational age (Table S2, Table S3). Notably, significant differences between the control group and both probiotic groups were also detected after removal of all probiotic-specific ASV’s from the analysis (Figure S3). This suggests that the observed changes are not solely a direct consequence of the bacteria added to the enteric community by oral probiotic supplementation.

Additionally, neonates receiving probiotic supplementation at T1 showed less inter-individual variation and thus a more homogenous microbiota composition compared to infants in the control group (Figure 1d). Although the latter effect was not detected at subsequent time-points (Figure S2b-f), the significant difference in the global microbial community structure remained also shortly (T2) and long (T3) after cessation of probiotic supplementation (Figure 1e & f). The Bray-Curtis dissimilarity between samples collected during probiotic supplementation (T1) and both follow-up time-points (T2 and T3) was significantly lower for both probiotic groups as compared to the control group (Figure 1g,h). This suggests that probiotic supplementation reduces the temporal compositional variation and results in a more stable microbial community structure.

### Probiotic administration influences specific taxa of the endogenous microbiota

**Figure 2.**
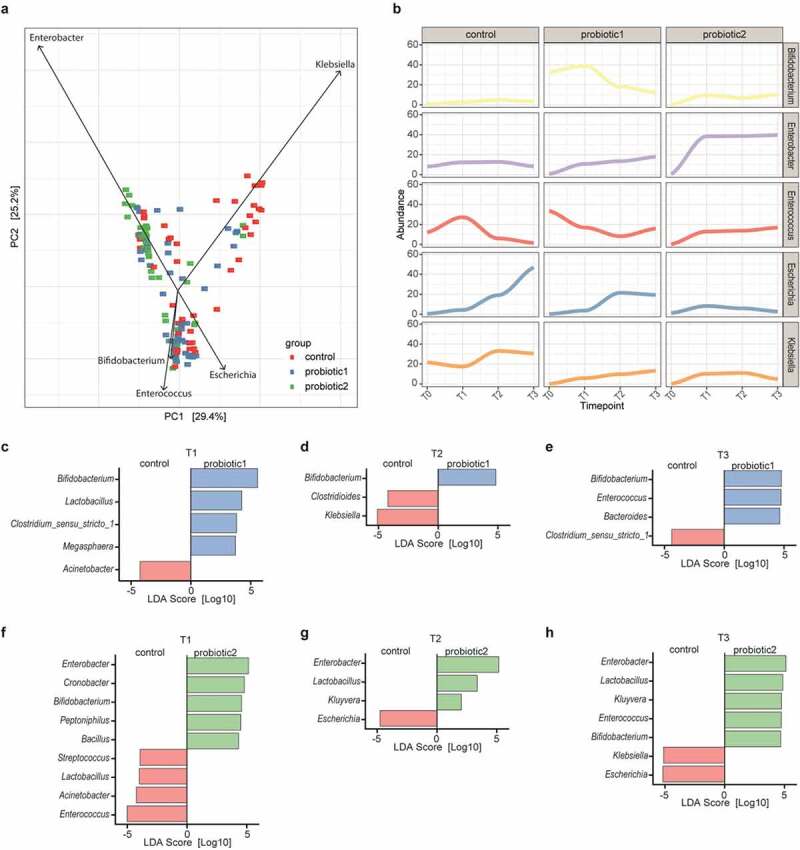
**Association of probiotic administration with specific taxa**. (a) Principal Component Analysis (PCA) illustrating the separation between the control and probiotic groups at T1-T3. Vectors depict the five genera that explained most of the variation in microbial community structure (b) Relative abundances of the five genera over time for the three different groups. (c) Linear discriminant analyses with Effect Size (LEfSe) were employed to identify differentially abundant bacterial genera between probiotic 1 supplemented and control neonates at T1, (d) at T2, (e) and at T3. (f) LefSe between probiotic 2 supplemented and control neonates at T1, (g) at T2, (h) and at T3 (LDA-score >0.2 and *p* < 0.05, Wilcoxon signed-rank test)

We subsequently assessed how probiotic administration influenced specific endogenous bacterial taxa over time (T1-T3). First, we determined how the genera that explained most of the variation in the overall microbiota community structure were associated with probiotic administration. *Klebsiella, Escherichia, Bifidobacterium, Enterococcus*, and *Enterobacter* represented the main genera driving the separation in microbial community structure between the three groups. Enhanced abundance of *Klebsiella* was typically found in neonates without probiotic supplementation. In contrast, neonates receiving P2 were characterized by higher abundance of *Enterobacter* and reduced abundance of *Escherichia* (Figure 2a). Increased relative numbers of *Bifidobacterium* and *Enterococcus* were most commonly observed among neonates that received P1.

Secondly, the taxonomic changes of these five genera over time indicated that probiotic supplementation reduced the temporal fluctuations in microbiota composition. Whereas the abundance of *Enterococcus, Escherichia*, and *Klebsiella* were highly dynamic in the control group, a more stable pattern could be observed in probiotic-supplemented neonates (Figure 2b). This more stable pattern was accompanied by an expansion of the genus *Bifidobacterium* in the P1 group and an expansion of the genus *Enterobacter* in the P2 group.

Lastly, linear discriminant analysis revealed that *Bifidobacterium* was the most strongly enriched genus among neonates receiving P1 at all time-points, while lactobacilli were only significantly enriched at T1 (Figure 2c-e). In addition, after probiotic supplementation neonates harbored less *Klebsiella* and two members of the Clostridiales order when compared to control neonates. Moreover, although the second probiotic similarly to P1 was associated with a higher abundance of bifidobacteria and decreased levels of *Klebsiella* at T3, these neonates could most profoundly be differentiated from the control group by a higher abundance of *Enterobacter* at all time points (Figure 2f-h). Remarkably, the neonates receiving P2 showed a strong decrease in *Enterococcus* and *Escherichia* at T1 and T2-T3, respectively. Furthermore, in both probiotic groups, the abundance of bifidobacterial species other than those included in the probiotic formulas was enhanced when compared to the control neonates (Figure S4a-f).

### Intestinal colonization by probiotic bacteria after cessation of supplementation

**Figure 3.**
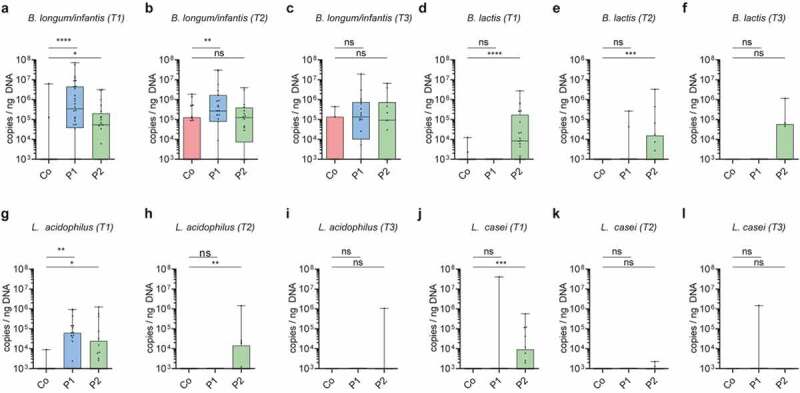
**Association of probiotic administration with the detection of probiotic species**. (a-l) Quantitative abundance (log10 copies/ng DNA) during probiotic supplementation at T1, after cessation of probiotic supplementation at T2 and T3 respectively of the probiotic bacteria *Bifidobacterium longum/infantis* (a-c), *Bifidobacterium lactis (d-f), Lactobacillus acidophilus* (g-i), *Lactobacillus casei* (j-l). (Kruskal-Wallis test with Dun’s test for posttest comparisons (control-group is reference), mean and SD; *, *p* < 0.05; **, *p* < 0.01; ***, *p* < 0.001;****, *p* < 0.0001; ns, not significant)

In order to quantitatively monitor the probiotic bacteria’s ability to colonize and persist after cessation of supplementation, we established species-specific qPCRs detecting the administered probiotic bacteria. Using these qPCRs, none of the probiotic bacteria were detected prior to supplementation (T0) (Figure S4g-j). In contrast, qPCR detection confirmed the markedly higher abundance of *B. longum/B. infantis* and *L. acidophilus* in neonates receiving P1 and *B. longum/B. infantis, B. lactis, L. acidophilus*, and *L. casei* in neonates receiving P2 as compared to controls (Figure 3a-l). Notably, *B. longum/B. infantis* was still enriched at T2 i.e., after cessation of supplementation with P1, whereas *B. lactis* and *L. acidophilus* remained increased after cessation (T2) of supplementation with P2. Importantly, probiotic bacteria did not permanently colonize since no significant differences between probiotic and control groups were observed at T3. However, *B. lactis* was still detectable in some (4/11) infants that received P2 at T3 (Figure 3f).

**Figure 4.**
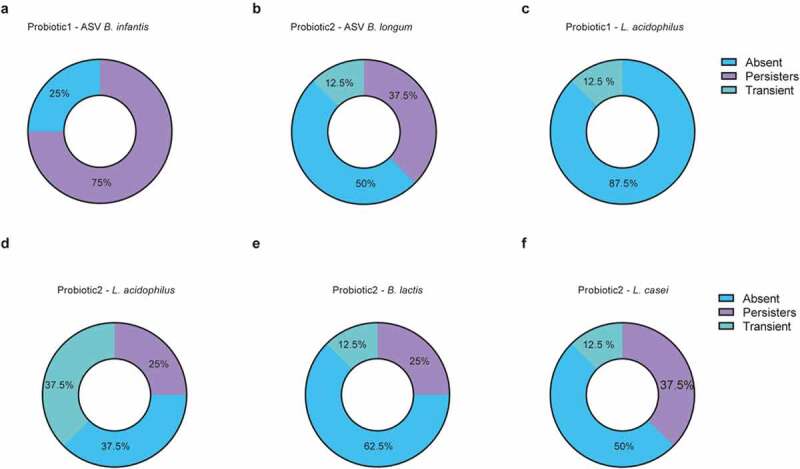
**Colonization by probiotic bacteria after cessation of supplementation**. (a-f) Colonization pattern from longitudinally sampled neonates (n = 8/group) after cessation of probiotic supplementation for probiotic specific ASV-tracked (a) B. infantis in probiotic 1 supplemented neonates; (b) B. longum in probiotic 2 supplemented neonates, (c) qPCR-tracked Lactobacillus acidophilus in probiotic 1 supplemented neonates; qPCR-tracked (d) Lactobacillus acidophilus, (e) Bifidobacterium lactis, (f) and Lactobacillus casei in probiotic 2 supplemented neonates. Categorized into ‘persistent’ colonizers (first two weekly timepoints after cessation were positive for probiotic strains), ‘transient’ (sample collected in the first two weeks after cessation was positive, but subsequent sample was negative) and ‘absent’ (first timepoint upon probiotic cessation was negative for probiotic bacteria)

To further investigate the nature and duration of probiotic persistence we analyzed fecal samples of 16 neonates collected weekly during and until 20 weeks after cessation of probiotic supplementation. Here we took advantage of the whole genome sequencing data generated from the cultured probiotic strains to identify the specific 16S rRNA gene V3-V4 region and to distinguish between *B. longum* subsp. *infantis* and *B. longum* subsp. *longum*. We then tracked probiotic *Bifidobacteria* using ASVs (i.e., ASVs that matched the WGS-sequences of the isolated probiotic strains) and performed qPCRs for all other probiotic bacteria (Figure S5). Since the prevalence pattern of the different probiotic bacteria varied considerably (Figure S5a-e), we categorized the neonates into ‘persistently’ colonized (first two samples after cessation positive for probiotic strains), ‘transiently’ colonized (at least one sample collected in the first 2 weeks after cessation positive for probiotic strains, but subsequent samples negative) and not colonized (1st and 2nd sample upon probiotic cessation negative for probiotic strains). Thereby, the probiotic bifidobacterium in P1 (*B. infantis*) was shown to colonize the neonatal gut persistently in the majority of children after cessation whereas this rate was reduced for *B. longum* in P2 (Figure 4 a-b). On the other hand, *L. acidophilus* was absent in almost all neonates that had received P1 whereas it remained present (persistently or transiently) in the majority of neonates supplemented with P2 (Figure 4c-d). Neonates supplemented with P2 also showed persistence of *B. lactis* and *L. casei* in some neonates, but most neonates had become negative for these probiotic strains (Figure 4e-f). In some children, the colonization of *Bifidobacterium* and *L. casei* persisted up to 15 weeks after cessation of probiotic supplementation (Figure S5a-e).

### Abundance of pathobionts and prevention of necrotizing enterocolitis

**Figure 5.**
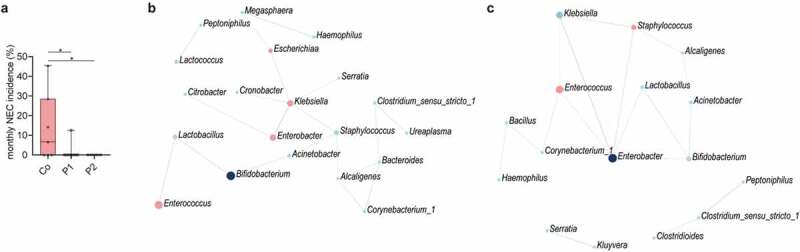
**Probiotic intake may prevent necrotizing enterocolitis by interfering with pathobionts**. (a) Monthly NEC-incidence during the 2-year study period among all preterm born neonates (n = 179, gestational age<32 weeks) stratified according to supplementation group. In total, 9, 1, and 0 NEC cases were diagnosed in the control group, P1 group and P2 group, respectively. (b) SparCC correlation network analyses with genera at T1 in the probiotic 1 supplemented, (c) and probiotic 2 supplemented neonates as compared to control neonates (correlation >0.3, *p* < 0.05). Blue circles indicate genera with an increased abundance in probiotic groups, while red circles represent genera enriched in control neonates. Red lines indicate negative correlations, while blue lines indicate positive correlations

Probiotic supplementation both increased the stability in the overall community structure of the neonatal gut microbiota as well as reduced the abundance of pathobionts known to induce strong proinflammatory responses. Since these changes might protect against the adverse effects of an altered microbiota, we next investigated the influence of probiotic supplementation on the incidence of necrotizing enterocolitis (NEC) and the presence of clinically relevant antimicrobial resistance (AMR) genes. Despite the low overall occurrence of NEC during our study period (n = 10), a significantly reduced incidence was observed in neonates supplemented with P1 or 2 as compared to control neonates (Figure 5a). We therefore analyzed the composition of the fecal microbiota of three NEC-patients prior to the clinical onset of the disease. Interestingly, a dramatic bloom of enterococci was observed, representing the most abundant taxa before the onset of the disease (Figure S6a). Notably, *Enterococcus* is only low abundant in neonates without NEC as depicted from the control group at T2 accompanied with *Klebsiella* as the most dominant taxa (Figure 2b). To further elucidate the role of the probiotic bacteria herein, we performed SparCC correlation network analyses taking the compositional nature of the data into account.^13^ Interestingly, probiotic supplementation with bifidobacteria and lactobacilli both correlated with the abundance of the NEC-pathobiont *Enterococcus*.^14,15^ In neonates receiving P1, bifidobacteria, and lactobacilli were positively correlated. Lactobacilli in turn exhibited a direct negative network correlation with enterococci (Figure 5b, Figure S7a). In neonates receiving P2, bifidobacteria, and lactobacilli positively correlated with *Enterobacter* that in turn exhibited a negative correlation with the NEC-pathobiont *Enterococcus* (Figure 5c, Figure S7b). No significant influence of probiotic supplementation was observed in this study on the clinical outcomes sepsis, oxygen supplementation, and parenteral feeding (Figure S8).

As probiotic administration was associated with a decreased abundance of *Enterococcus, Escherichia* and *Klebsiella* in preterm infants (Figure 2c-h), probiotics might also reduce the prevalence of antibiotic-resistant strains. The presence of resistant strains significantly hampers an effective antimicrobial therapy in the event of infection. Enterococci can harbor the resistance genes *vanA* and *vanB* conferring resistance to glycopeptide antibiotics such as vancomycin and teicoplanin. Enterobacteriaceae, such as *Escherichia* and *Klebsiella*, can encode for extended-spectrum beta-lactamases (ESBLs) conferring resistance to most beta-lactam antibiotics. We therefore investigated the presence of vanA and *vanB* as well as the most prevalent ESBL (*CTX-M* group 1, 2, and 9) genes. However, the prevalence of these antibiotic resistance genes was not significantly altered by probiotic supplementation, possibly due to the low overall presence of antibiotic resistance genes in the enteric microbiota of the population analyzed (Figure S6b-c).

## Discussion

With weekly fecal sampling and clinical monitoring, our study represents a longitudinal observational study with three groups, a group of children that was left untreated (control group) and two groups of children that received two different probiotic supplements (P1, and P2). Although the different groups were sampled during consecutive time periods, the standard medical care regimen (except probiotic supplementation), the medical and nursing staff as well as the neonatal intensive care unit (NICU) environment and medical equipment were identical for all patients included in the study. Notably, microbial community structure analysis between the consecutive time periods did not detect significant seasonal effects or cross contamination by probiotic strains or bacteria of the NICU environmental (Fig. S9).^16,17^

In contrast to many previous studies among adults and infants,^11,18,19^ we showed persistence of several probiotic strains upon cessation of supplementation. Administered *B. infantis* persisted for 3–15 weeks following cessation in approximately three quarter of the neonates, whereas persistence of lactobacillus strains was observed less frequently. This superior persistence of bifidobacterial strains when compared to lactobacilli has also been observed in several other studies.^10,12,17^ The mechanisms underlying the differential persistence are largely unclear, but the gut luminal milieu, the characteristics of the administered bacterial strains as well as their competitive fitness may all contribute. Interestingly, we detected differences in the abundance of the probiotic bacteria between our two supplemented probiotic formulas, which suggests species or strain differences or an influence by the co-administered probiotic strains. For example, *B. lactis* but not *B. longum* remained significantly enriched after cessation of supplementation in neonates that received P2 when compared to neonates in the control group. In addition, *L. acidophilus* persisted in neonates that received P2 in contrast to P1 supplemented neonates.

Previous reports demonstrated that breast‐milk exerts a bifidogenic effect.^1,20^ This effect is to a large extent driven by maternal human milk oligosaccharides (HMOs), structurally diverse unconjugated glycans that are highly abundant in human milk but absent in formula nutrition. HMOs play a prebiotic role and promote the growth of *Bifidobacterium*.^21^ Breastfeeding might thereby also promote the colonization with bifidobacterial strains administered as probiotics. This could explain the marked colonization by the administered probiotic *B. infantis* strain, a known to utilizer of HMOs in our study.^22^ Whereas the size of the study population did not allow the analysis of a possible association between breastfeeding and colonization efficacy of probiotic bacteria in the present study, future investigations should address this interesting and clinically relevant question.

Probiotic administration significantly altered the enteric microbiota composition even after removing probiotic ASVs from the sequencing data. Thus, the administered probiotic bacteria directly or indirectly influence the endogenous microbiota consistent with the results of other studies.^10–12,17^ Most notably, although bacterial richness and diversity remained unaffected, probiotic administration significantly reduced the inter-individual variation and temporal microbiota changes. Neonates, in particular preterm neonates, have been shown to exhibit high inter-individual variation and major fluctuations in the enteric microbiota composition during the early postnatal period.^1,4,23,24^ High individual variation and compositional fluctuations may contribute to a decreased resilience and colonization resistance, and thus a more stable microbiota composition might protect from disease-promoting compositional changes. Moreover, the preterm microbiota is typically dominated by pathobionts such as *Escherichia, Enterobacter*, and *Klebsiella* that produce potent immunomodulatory molecules such as endotoxin and fimbriae and frequently carry genes conferring antibiotic resistance.^3,14,25^ Reducing such bacteria might lower the risk of inappropriate inflammation or systemic infection. In this respect, *Klebsiella*, one of the major bacteria driving the global community structure in the preterm neonates without probiotic supplementations did not contribute to the overall community structure in children supplemented with probiotics administration. An observation which is in line with the results of the study by Alcon-Giner and colleagues.^17^

As expected probiotic supplementation led to enrichment of *Bifidobacterium spp*. and *Lactobacillus spp*. However, this was not limited to an enrichment of the probiotic bifidobacterial and lactobacillus species. In accordance with another recent report, the administration of probiotic bifidobacterial species fostered the growth of endogenous bifidobacterial species such as *B. breve, B. bifidum* and *B. animalis*.^17^ Cross-feeding between species within a genus or induced adaptation of the local gut environmental might contribute to this. In contrast, pathobionts such as *Klebsiella* or *Escherichia* as well as *Clostridioides* and enterococci were reduced in probiotic-treated infants. These results are largely consistent albeit not identical with observations described in other studies.^10,17^ Despite the reduced abundance of enterococci and Enterobacteriaceae (*Klebsiella* and *Escherichia*), the prevalence of genes encoding vancomycin resistance and ESBLs was not reduced among neonates supplemented with probiotics. These findings contrast a previous study, which observed ESBL-genes in stools of non-probiotic very preterm infants but not in probiotic-treated extremely preterm infants.^26^ Previous clinical and animal studies suggested that the enteric microbiota together with the immature mucosal tissue contribute to the etiology of NEC.^25^ Changes in the microbiota composition would therefore be expected prior to disease onset. A number of human cohort studies with regular postnatal sampling of preterm neonates reported on the enteric microbiota composition in healthy infants and infants with NEC.^14,27^ However, no specific pathogen, pathobiont, or microbiota signature was detected that unequivocally predicted disease onset. Notably, almost all cases of NEC documented during the study period occurred in neonates of the control group confirming a significant protective effect of probiotic supplementation. For three patients with NEC, consecutive samples were available prior to disease onset. All three exhibited a significantly enhanced abundance of enterococci prior to disease onset. An increase in enterococci or enterococcus-associated secondary metabolite gene clusters was also noted in other studies.^14,28^ Additionally, certain strains of the *Enteroccus faecalis* have been shown to increase the tissue pathology in a rodent NEC model.^15^ Two of the patients in the present study also exhibited an enhanced abundance of *Klebsiella*, an organism associated with an increased risk of NEC in several studies.^14,27^ Moreover, an increased abundance of Enterobacteriaceae prior to NEC onset has been found.^27,29,30^ As expected, the abundance of members of the Enterobacteriaceae family (*Escherichia* and *Klebsiella*) also increased in abundance during the NEC-induced intestinal inflammation.^27^ Although the mechanism by which probiotics protect against NEC is unclear, our results are consistent with the idea that it may include enhanced resilience of the enteric microbiota to exogenous stimuli, the reduction of pathobionts or indirect immunomodulatory effects on the host’s developing intestinal tissue and mucosal immune system.^31,32^

The daily dose of 10^9^ supplemented bacteria is substantial given that the total bacterial number within the newborn gut is estimated at 10^6^–10^12.33,34^ Given the immature intestinal tissue and mucosal immune system and high susceptibility to systemic bacterial infection of preterm neonates a careful consideration of potential risks of oral administration of viable bacteria is warranted. Cases of systemic infections with orally applied probiotic bacteria in extremely preterm neonates have been reported.^35^ In the present study no adverse effects or infections by the administered probiotic were observed during the study period.

Nevertheless, the observational character represents a limitation of our study precluding any blinded and randomized study design. The consecutive “enrollment” of the three patient groups also harbors the risk of unidentified changes in the standard patient care during the study period that may influence the clinical outcome. In addition, the relatively small sample size limits the statistical power of our analysis in particular with respect to the analysis of the enteric microbiota prior to the clinical onset of NEC. Altogether our study provides important insights that warrant confirmation, preferably in experimental studies. Sampling after discharge would ideally be included in such future studies in order to assess the long-term consequences of probiotic supplementation.

In conclusion, our results show a significant influence of oral probiotic administration on the global microbial community structure and the abundance of specific taxa. The data emphasize the unique period of early infancy for the establishment of the enteric microbiota and suggest a beneficial effect of probiotic supplementation to preterm neonates.

## Patients and methods

### Study design

Preterm infants of gestational age <32 weeks were included in this natural experiment at the NICU of RWTH Aachen University Hospital between January 2016 and 2018. Initially, from birth until 36 weeks of gestational age, infants received daily a supplemented milk regimen (Infloran, 250 mg/day) as a standard procedure (P1 group). The probiotic Infloran contained the bacteria *Lactobacillus acidophilus* (ATCC 4356) and *Bifidobacterium longum* subspecies *infantis* (ATCC 15697) at a 1:1 ratio (each strain at 10^9^ CFU/250 mg). This standard supplementation with probiotic bacteria had to be temporarily suspended due to the unavailability of this probiotic on the market. During this time between May and December 2016, preterm neonates only received milk without probiotic supplement (control group). After December 2016, probiotic administration to all preterm (<36 weeks) neonates was reinstalled with another probiotic supplement (DarmfloraPlus, 250 mg/day) containing *Lactobacillus acidophilus* La-14 (ATCC SD5215), *Bifidobacterium longum* subsp. *longum* Bl-05 (ATCC SD5588), Lactobacillus *casei* Lc-11 (ATCC SD5213) and *Bifidobacterium animalis* subsp. *lactis* with equal numbers for all four strains (each strain at 10^9^ CFU/250 mg). From the entire population of preterm neonates admitted during the study period, samples were selected based on the first available sample within the time window described in the infographic in Figure 1a. Thus, T0 only includes samples that were collected prior to the start of the probiotic supplementation. As these samples could not always be obtained, the number of children with a sample at T0 is smaller as compared to the number of children in other time windows. Also for the subsequent time windows (T1, T2, T3), we selected samples that were collected within the specified time window (i.e., 3–4 weeks after birth for T1, 7–9 weeks after birth for T2 and 10–12 weeks after birth for T3). If samples were available for the specified time window we subsequently checked if samples fulfilled the selection criteria for the given time window (probiotics still being administered for T1, probiotic supplementation ceased for T2 and T3). As a result and because of missing samples or because samples did not fulfill the selection criteria, the number of samples/children per time window vary slightly.

With one or more *Lactobacillus spp*. and one or more *Bifidobacterium spp*, both probiotic formulation fulfill the criteria of a probiotic regimen with moderate to high-quality evidence of reduced all-cause mortality as recently shown in a large metaanalysis.^7^ The viability of the probiotic strains within the two products and the absence of contaminating bacteria were confirmed by culture; the identity of the respective bacterial strains was confirmed by sequencing. Sequencing also confirmed the different subspecies of the two *Bifidobacterium longum* strains (subsp. *longum* and subsp. *infantis*) and the close relatedness of both *L. acidophilus* strains (ATCC 4356 and ATCC SD5215). The sequences are available upon reasonable request. There were no exclusion criteria for the probiotic supplementation. NEC and sepsis were diagnosed based on the modified Bell’s score and clinical as well as laboratory parameters, respectively, in accordance with the national guidelines of the German Society of Neonatology and Pediatric Intensive Care Medicine.^36–38^ Spontaneous intestinal perforation was excluded based on the clinical presentation and histological report. The initial antibiotic standard regimen for neonates consisted of ampicillin (100 mg/kg) and tobramycin (3 mg/kg) in 3–4 daily doses. No indomethacin or proton pump inhibitor (PPI) was administered in accordance with the national guidelines. Infants were sampled weekly during their hospitalization on the neonatal intensive care unit (NICU). The final samples were selected based on the first available sample within the timepoints described in Figure 1a. In an additional group of 16 neonates, we longitudinally sampled weekly from supplementation up to 20 weeks after cessation of the probiotic to assess the persistence. Moreover, we also included longitudinal samples of three patients that developed NEC closely to the diagnosed onset of the disease. The study was recorded in the German Register Clinical Studies under accession number DRKS/GCTR 00021034 and was approved by the ethics committee of the medical faculty of the RWTH University of Aachen (approval number EK307/15).

### Culture, isolation, and whole-genome sequencing of probiotic strains

Serial tenfold dilutions were made of each of the probiotic products dissolved in phosphate-buffered saline (PBS) and 100 µL of dilutions ranging from 10^6^–10^2^ CFU/mL were plated onto Columbia blood agar plates with sheep blood (Oxoid, Basingstoke, UK) and incubated anaerobically at 37°C for 48–72 h. Morphologically different colonies were isolated and identified by whole genome sequencing. In brief, pure cultures suspensions of 0.5 mL were made in PBS for total DNA isolation using MasterPure complete DNA and RNA purification kit (Epicenter, MC 85200), following the manufacturer’s protocol. Sequencing libraries were prepared using Illumina’s NexteraXT kit. Whole genome sequencing was performed on an Illumina MiSeq by 2 × 250 paired end sequencing using v2 flowcell. *De Novo* assembly was performed using Skesa (setting: with_paired_ends).^39^ Subsequently, strain identification was performed screening all contigs of the *de novo* assembly using Basic Local Alignment Tool (BLAST) from NCBI (accessed July 2020).

### Microbial profiling of fecal samples

Metagenomic DNA was isolated from fecal samples thawed on ice and resuspended in 600 μL DNA stabilization buffer (Stratec Biomedical) following a previously described protocol.^40^ Briefly, 500 µl 5% (w/v) N‐laurolylsarcosine and 250 µl 4 M guanidinethiocyanate were added to the samples and cells were mechanically lysed by repeated bead beating (3 x 40 s) with 500 mg 0.1 mm glass beads (Roth). Next, samples were vortexed and centrifuged (15.000 × g for 3 min at 4°C) after adding 15 mg poly‐vinylpolypyrrolidone. Subsequently, the supernatant was incubated with 1% ribonuclease (10 mg/mL) for 40 min at 37°C. Finally, column-based purification was performed using the NucleoSpin gDNA Clean‐up Kit (Macherey‐Nagel). The bacteria-specific primers 341 F and 785 R were used to amplify the variable 3–4 region of the 16S rRNA gene from 12 ng metagenomic DNA by a two-step PCR (2x 15 cycles).^41,42^ PCR amplicons were purified using the AMPure XP system (Beckmann), and sequenced using 2 × 275 paired-end reads spiked with 25% PhiX on a Miseq platform (Illumina Inc.). All generated sequences from this study have been deposited to the Qiita and ENA databases and can be accessed under No. 13071.

### Microbiome data processing and statistics

A total of 8,335,421 generated sequences were processed using the DADA2 package in R to create single amplicon sequence variants (ASV’s).^43^ Quality filtering, removing of sequence errors, and chimeras was performed using default settings and taxonomy was assigned with SILVA on a species level. For the removal of potential contamination, we used the Decontam package in R with the ‘combined’ setting.^44^ Here, all samples with a DNA concentration below the detection limit (<0.10 ng/ul) were set at half the lowest detected concentration of 0.05 ng/µl and extraction controls were used as negative controls. Subsequently, we omitted samples with a low sequencing depth (<8000 sequences) and removed ASVs that were low abundant (<0.25% in all samples). This resulted in a total of 7,708,120 sequences ranging from 7,727 to 72,816 reads per sample (median 21,113) that belonged to 4,981 ASVs for downstream analyses. Lastly, we normalized the retained ASV counts by dividing each value to the total sum per sample and multiplied by the lowest sample depth.^45^

We computed the alpha-diversity (Shannon index and observed species) and the Bray-Curtis metrics in order to determine the microbial community structure using the R package Phyloseq 1.30.0.^46^ The ordination of samples based on the Hellinger transformed genus composition or Bray-Curtis dissimilarity was visualized using Principal Component Analysis (PCA) or Principal Coordinate Analysis (PCoA), respectively. The Mann–Whitney U or Kruskal–Wallis tests were used to test for differences in relative abundances, Bray-Curtis distances, microbial richness and diversity between two or multiple groups, respectively. Following the Kruskal–Wallis test, the Dunn’s test was performed for pairwise post hoc comparisons. Subsequently, the alpha-diversity was used as a continuous variable in a linear regression model to test for a significant trend across timepoints. In order to evaluate significant separations between groups of samples, we performed a permutational multivariate analysis of variance (PERMANOVA) based on Bray-Curtis distances. We examined factors that were significantly associated with the microbial community variation at the different timepoints by PERMANOVA to determine potential confounders: timepoint, feeding-type, sepsis, antibiotics, mode of delivery, birthweight, gestational age, sex, and PROM. We next combined the identified potential confounding factors with the variable probiotic group in a multivariate analyses and reevaluated the significance. Moreover, we also tested the dispersion of the samples, a required assumption for the PERMANOVA test. All these statistical analyses were performed two-sided with R 3.6.1 or GraphPad Prism 8.

SparCC correlation analyses were performed to identify specific associations taking the compositional nature of the data into account by MicrobiomeAnalyst with default settings.^47^ Only correlations of >0.3 that were statistically significant were included in the network analyses. Differentially abundant bacterial genera were identified using Linear discriminant analysis Effect Size (LEfSe) with default settings.^48^

### Detection of probiotic bacteria & antimicrobial resistance genes (qPCR)

For the quantification of *Lactobacillus acidophilus* (5ʹ AAACTGCAATTTAAGATTATGAGT TTC/GGTACCGTCTTGATTATTAGTGTA 3ʹ), *Bifidobacterium lactis (*5ʹ CATCGCAACTTCA CCCACATTG 3ʹ/5ʹ ATGCCGTACCCCT GAATGAAG 3ʹ), *Bifidobacterium longum* (5ʹ CGGCGTYGTGACCGTTGAAGAC 3ʹ/5ʹ TGYTTCGCCRTCGACGTCCTCA 3ʹ), *Lactobacillus casei* (5ʹ TGCCCATTAGCA TACTGGACC 3ʹ/5ʹ ACCCGAGCCTTTGCCAA 3ʹ) all fecal samples were subjected to real-time PCRs.^49,50^ We validated the existing assays by amplifying the DNA of the cultured probiotic strains. Upon confirmation of the PCR amplicons by Sanger sequencing, products were cloned into a pGEM-T easy vector (Promega Corporation, Madison, WI, USA) for construction of standard curves. Thereafter, the amount of plasmid copies normalized to the amount of DNA were calculated for each fecal sample from the cycle threshold values using the standard curves. For this assay 2 µl DNA and 0.4 μM primer was used as input, employing SYBR Green chemistry (Supermix™ SYBR®, Bio-Rad) and a CFX96 System (Bio-Rad). All targets were amplified at an annealing temperature of 65°C except for the *Lactobacillus acidophilus* (60°C). CFX Manager 3.1 (Bio-Rad) was used for analysis of the real-time qPCR data. Samples that were below the detection limit were included with zero plasmid copies.

The qPCR of antimicrobial resistance genes vanA, vanB, CTXM-1, CTXM-2 was performed in a multiplex reaction and CTXM-9 in a simplex reaction as described earlier.^51,52^ Primer-probe concentrations for vanA (GCCGGAAAAA GGCTCTGAA/TCCTCGCTCCTCTGCTGAA) and vanB (CGCAGCTTGCATGGACAA/GGCG ATGCCCGCATT) were 800 nM of each primer and 200 nM probe (vanA_probe: FAM-ACGCAGTTATAACCGTTCCCGCAGACC-BHQ1/vanB_probe: VIC-TCACTGGCCTAC ATTC-MGB-NFQ). The qPCR of CTX-M (ATGTGCAGYACCAGTAARGTKATGGC/ATC ACKCGGRTCGCCNGGRAT) contained 500 nM of each primer and 100 nM probe (CTX-M-1: JOE-CCCGACAGCTGGGAGACGAAACGT-BHQ1/CTX-M-2: FAM-CAGGTGCTTATCGCTCTC GCTCTGTT-BHQ1/CTX-M-9: JOE-CTGGA TCGCACTGAACCTACGCTGA-BHQ1). Each PCR was performed in a total volume of 25 μL consisting of 12.5 μL ABsolute QPCR ROX Mix (Thermo Scientific, Waltham, MA, USA), the primer-probe mixture and template DNA. The amplification was achieved on a 7900HT Fast Real-Time PCR System (Applied Biosystems, Foster City, CA, USA) according to the standard conditions.

## Supplementary Material

Supplemental Material
