# Supplementary material for: Influence of probiotic supplementation on the developing microbiota in human preterm neonates
Source: Gut Microbes. 2020 Oct 23;12(1):1826747. doi: 10.1080/19490976.2020.1826747 (PMC7588225; doi:10.1080/19490976.2020.1826747)
Supplement: Supplemental Material [file KGMI_A_1826747_SM4232.zip › Supplementary information/Supplementary legends.docx]

**Figure S1: Microbiota diversity in preterms with or without probiotic supplementation. (A-D)** Richness (number of ASVs) of the three different groups at T0 **(A)**, T1 **(B)**, T2 **(C)** and T3 **(D)**. **(E-H)** Shannon index for the microbiota of the three different groups at T0 **(E)**, T1 **(F)**, T2 **(G)** and T3 **(H)**. Kruskal-Wallis test to controls with Dun’s post-test (control-group is reference), mean and SD; ns, not significant).

**Figure S2: Microbial composition and stabiliy in preterms with or without probiotic supplementation. (A)** PCoA based on ASV-derived Bray-Curtis dissimilarity does not show significant differences in microbial community structure between control and probiotic groups at T0 (not significant, Permanova)**. (B-C)** Bray-Curtis within group (control-group is reference)distance at T2 **(B)**, and T3 **(C)**. **(D-F)** PCoA based on ASV-derived Bray-Curtis dissimilarity in microbial community structure between T1-T3 for the control group **(D),** probiotic 1 group **(E)**, and probiotic 2 group **(F)**. (Kruskal-Wallis test with Dun’s test for post-hoc comparisons, mean and SD; ***, p<0.001; ns, not significant).

**Figure S3: Microbial composition in preterms with or without probiotic supplementation upon removal of probiotic ASVs**. **(A-D)** PCoA based on Bray-Curtis dissimilarity at T0 (ns: not significant, Permanova) **(A)**, T1 (p<0.001, Permanova) **(B)**, T2 (p<0.001, Permanova) **(C)** and T3 (p<0.001, Permanova) **(D)**.

**Figure S4: Association of probiotic administration with specific taxa. (A-C)** LefSe with bifidobacterial species between probiotic 1 supplemented and control neonates at T1, **(A)** at T2, **(B)** and at T3 **(C)**. **(D-F)** LefSe with bifidobacterial species between probiotic 2 supplemented and control neonates at T1, **(D)** at T2, **(E)** and at T3 **(F)** (LDA-score>0.2 and p<0.05, Wilcoxon signed-rank test). **(G-J)** Quantitative abundance before probiotic supplementation at T0 of the probiotic bacteria *Bifidobacterium lactis* **(G)**, *Bifidobacterium longum* **(H)**, *Lactobacillus acidophilus* **(I)**, *Lactobacillus casei* **(J)** (Kruskal-Wallis test with Dun’s test for post-hoc comparisons (control-group is reference), mean and SD; ns, not significant).

**Figure S5: Colonization by probiotic bacteria after cessation of supplementation. (A-E)** Colonization prevalence from longitudinally sampled neonates (n=8 / group) after cessation of probiotic supplementation for probiotic specific ASV-tracked *B. infantis* **(A)**, probiotic specific ASV-tracked *B. longum* **(B),** qPCR-tracked *Lactobacillus acidophilus* **(C)**, qPCR-tracked *Lactobacillus casei* **(D)**, and qPCR-tracked *Bifidobacterium lactis* **(E)**, No necrotizing enterocolitis, sepsis or antibiotic-treatment at the time of sample collection occurred in the included neonates directly after probiotic cessation except for P41 and P188 (both antibiotics provided 1-2 weeks after probiotic cessation for sepsis treatment).

**Figure S6: Microbial dynamics in preterm neonates developing necrotizing enterocolitis. (A)** The relative abundances of the 10 most abundant genera over time around the diagnosed onset of necrotizing enterocolitis (blue line) for three patients. **(B)** Prevalence of *vanA*/*vanB* genes conferring resistance to vancomycin. **(C)** Prevalence of extended-spectrum beta-lactamase encoding genes (CTX-M genogroups 1, 2 and/or 9) conferring resistance to most beta-lactam antibiotics.

**Figure S7: Correlations between genera upon probiotic administration. (A-B)** Correlation heatmap based on the coefficients of the SparCC analyses between relative genus abundances of control and probiotic 1 administered neonates at T1 **(A)**, or between control and probiotic 2 administered neonates at T1 (correlation>0.3, p<0.05) **(B)**.

**Figure S8: Clinical outcome in preterm neonates receiving probiotic supplementation. (A)** Frequency of diagnosed sepsis (%) in neonates that received no probiotic supplementation (Co) or supplementation with probiotic 1 (P1) or 2 (P2) including T1-T3. **(B)** Frequency of oxygen supplementation (>21%) in neonates that received no probiotic supplementation (Co) or supplementation with probiotic 1 (P1) or 2 (P2) including T1-T3. **(C)** Prevalence of neonates that received additional parenteral feeding (oral feeding < 100%) in the cohort on T1-T3 (ns; not significant, chi-squared test).

**Figure S9: Dissimilarity in microbiota community structure of control children as compared to children receiving probiotic supplementation.** Scatter plot of Bray-Curtis dissimilarity plotted against the time since termination of probiotic1 **(A-D)** or initiation of probiotic2 **(E-H).** The solid line depicts the linear regression line fitted to the data and the dashed lines depict 95% confidence intervals at T0 (R^2^=0.04236, slope = 0.0005629, p=0.4618) **(A),** T1 (R^2^=0.004502, slope = 0.0001081, p=0.1222) **(B)**, T2 (R^2^=0.003300, slope = 0.0001183, p=0.2640) **(C)** and T3 (R^2^=0.04236, slope = 4.556e-005, p=0.9032) of probiotic1 **(D)**. For probiotic2, these linear regressions are shown for T0 (R^2^=0.009396, slope = 0.001501, p=0.6305) **(E)** , T1 (R^2^=0.001361, slope = 0.0001253, p=0.4508) **(F)**, T2 (R^2^=0.007320, slope = 0.0002469, p=0.1175) **(G)** and T3 (R^2^=0.02572, slope = 0.0006796, p=0.1636) **(H)**.
