# Supplementary figures and images for: Influence of probiotic supplementation on the developing microbiota in human preterm neonates
Source: Gut Microbes. 2020 Oct 23;12(1):1826747. doi: 10.1080/19490976.2020.1826747 (PMC7588225; doi:10.1080/19490976.2020.1826747)

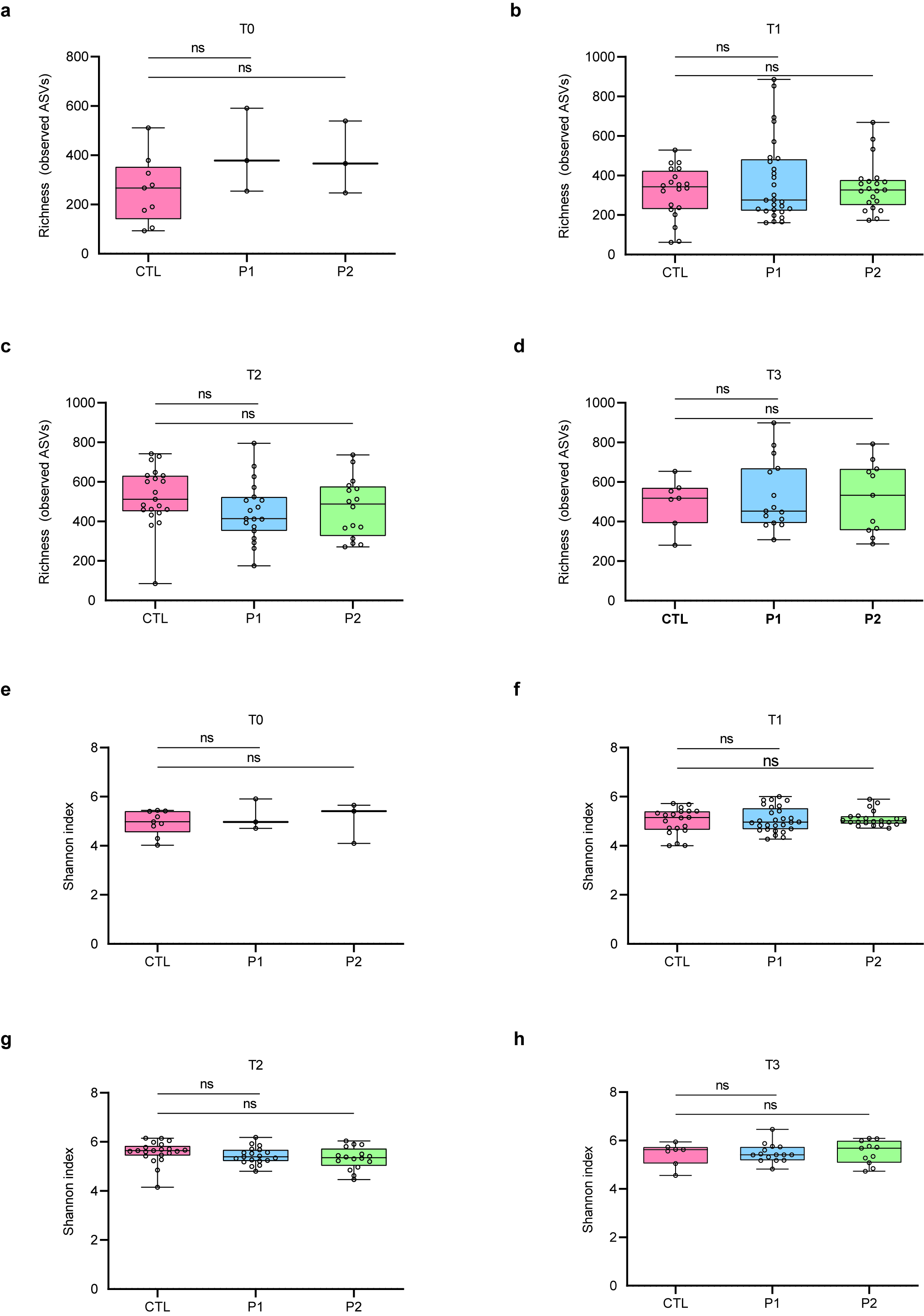

Supplement: Supplemental Material [file KGMI_A_1826747_SM4232.zip › Supplementary information/FigureS1.tif]

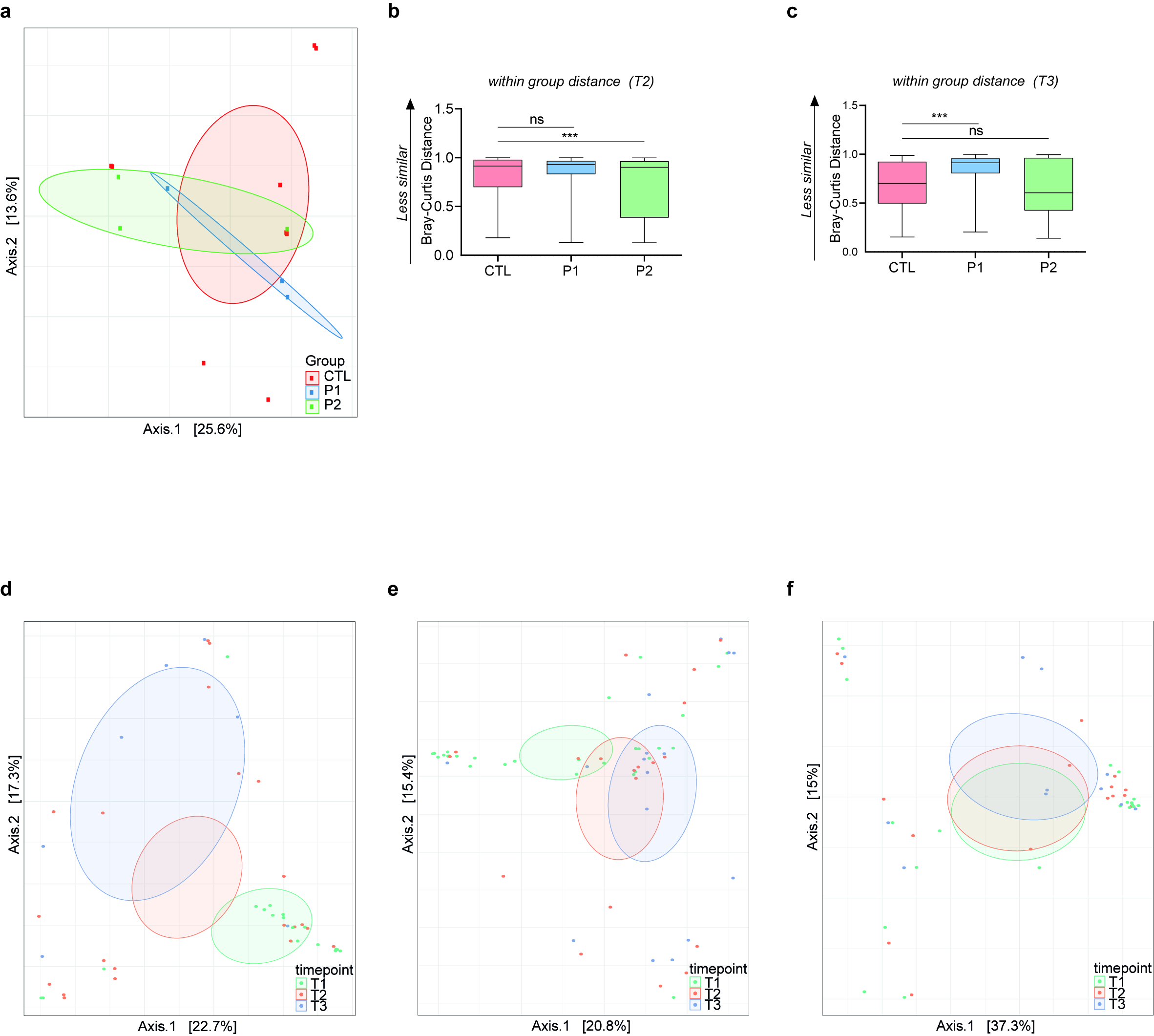

Supplement: Supplemental Material [file KGMI_A_1826747_SM4232.zip › Supplementary information/FigureS2.tif]

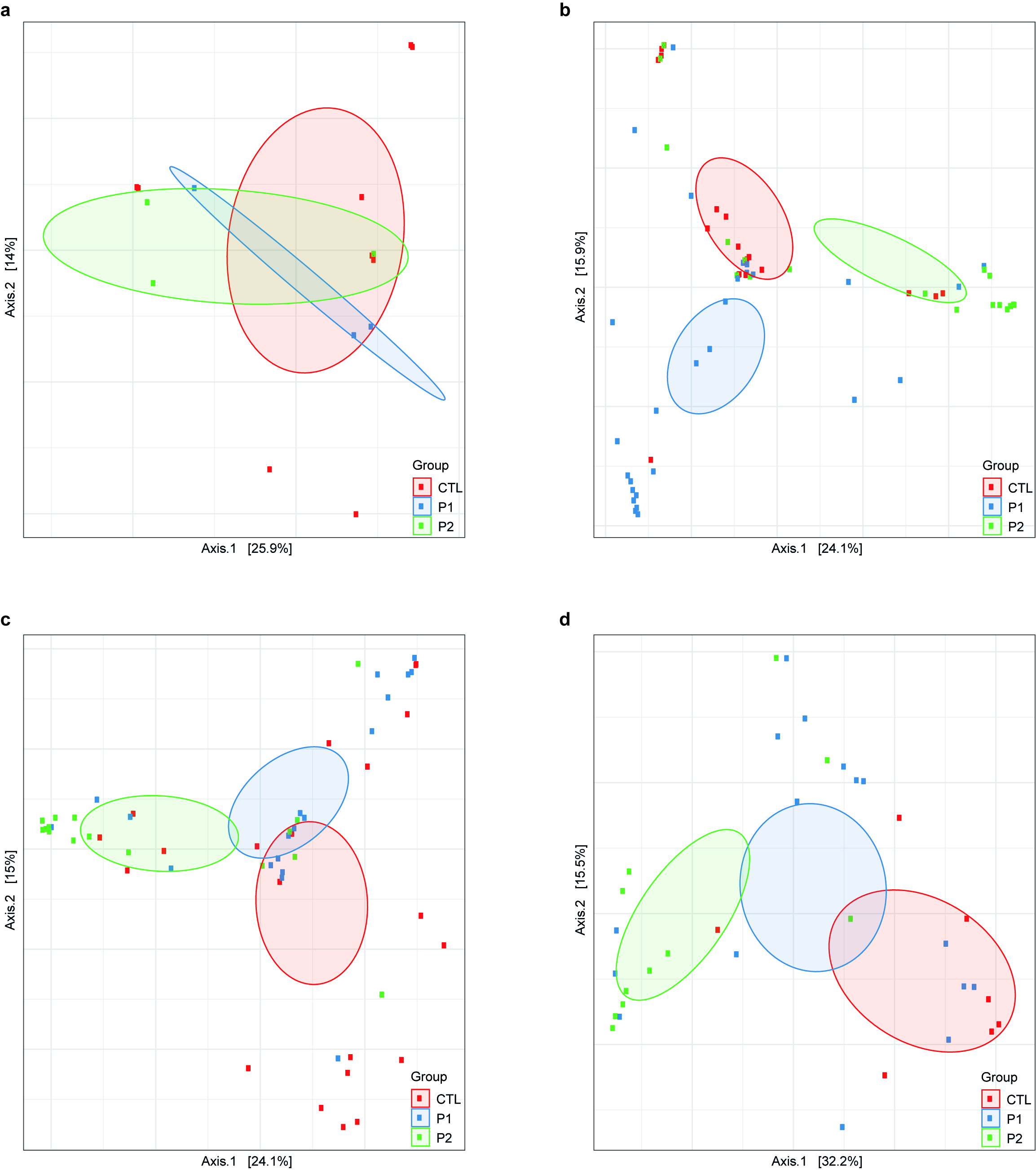

Supplement: Supplemental Material [file KGMI_A_1826747_SM4232.zip › Supplementary information/FigureS3.tif]

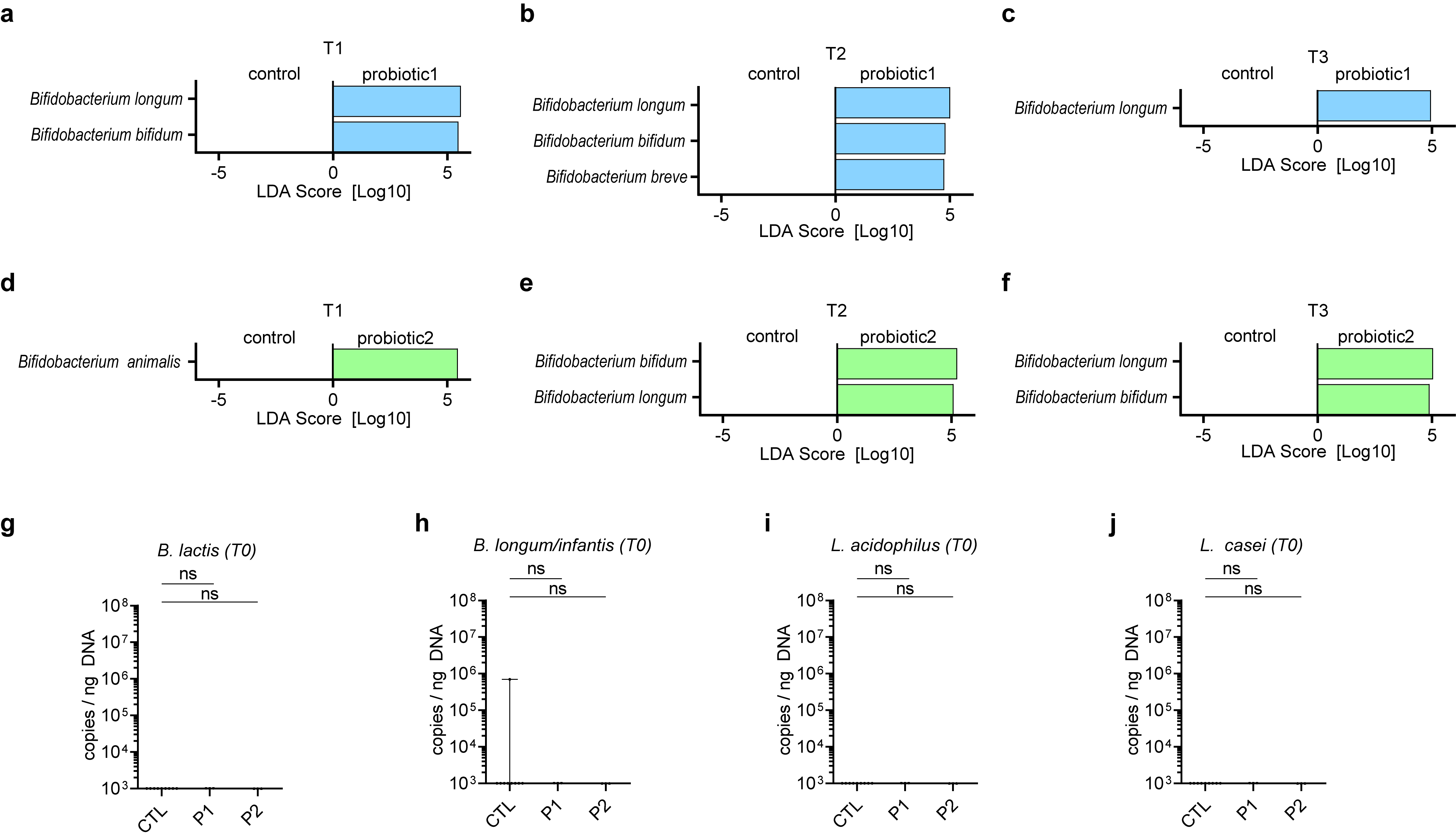

Supplement: Supplemental Material [file KGMI_A_1826747_SM4232.zip › Supplementary information/FigureS4.tif]

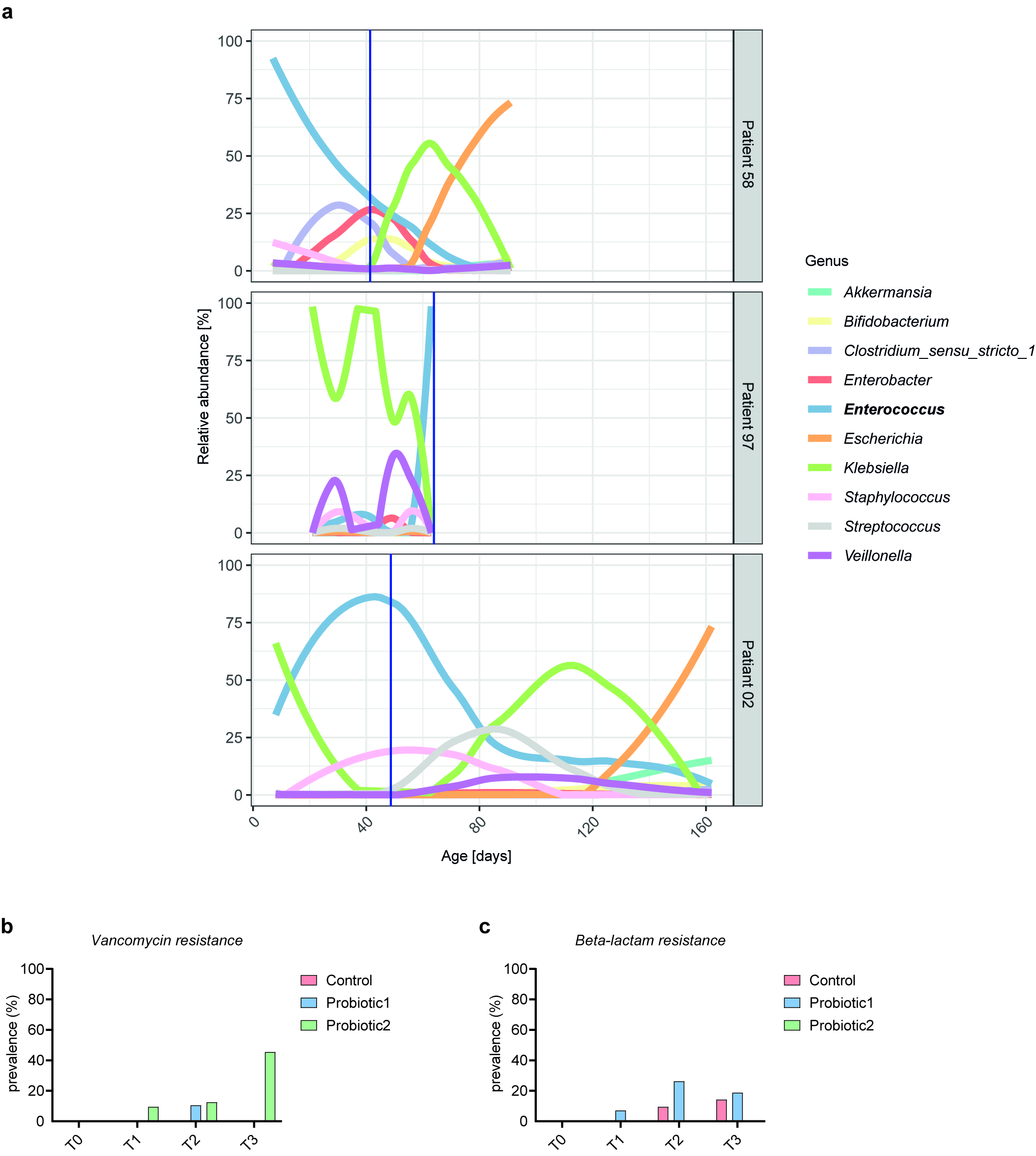

Supplement: Supplemental Material [file KGMI_A_1826747_SM4232.zip › Supplementary information/FigureS6.tif]

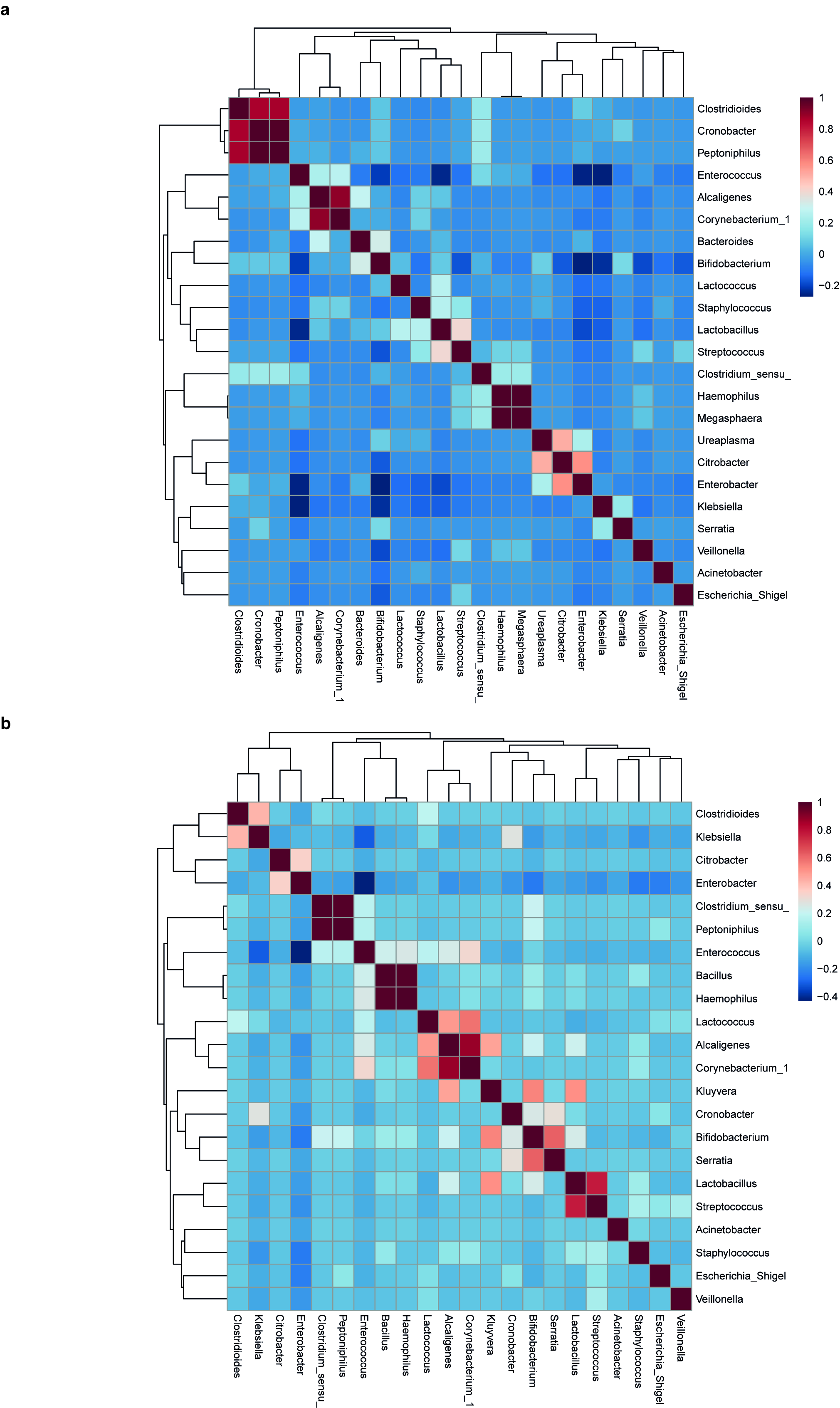

Supplement: Supplemental Material [file KGMI_A_1826747_SM4232.zip › Supplementary information/FigureS7.tif]

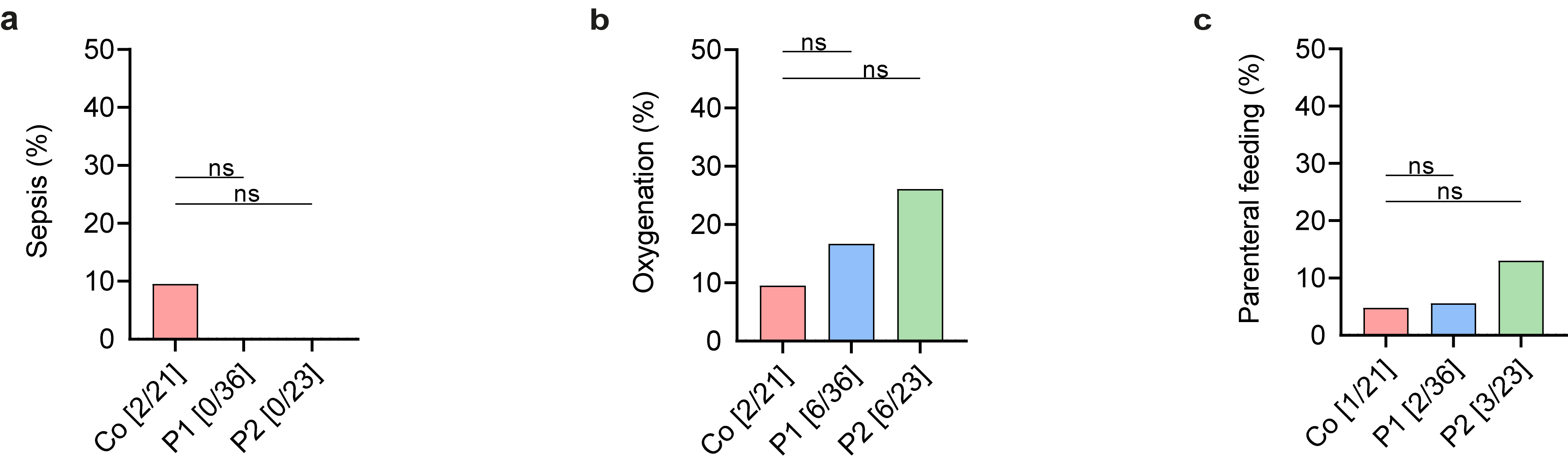

Supplement: Supplemental Material [file KGMI_A_1826747_SM4232.zip › Supplementary information/FigureS8.tif]
